# T-Cells Expressing a Highly Potent PRAME-Specific T-Cell Receptor in Combination with a Chimeric PD1-41BB Co-Stimulatory Receptor Show a Favorable Preclinical Safety Profile and Strong Anti-Tumor Reactivity

**DOI:** 10.3390/cancers14081998

**Published:** 2022-04-14

**Authors:** Nadja Sailer, Ina Fetzer, Melanie Salvermoser, Monika Braun, Doris Brechtefeld, Christian Krendl, Christiane Geiger, Kathrin Mutze, Elfriede Noessner, Dolores J. Schendel, Maja Bürdek, Susanne Wilde, Daniel Sommermeyer

**Affiliations:** 1 Medigene Immunotherapies GmbH, 82152 Planegg, Germany; nadja.sailer@gmail.com (N.S.); fetzer.ina@gmail.com (I.F.); m.salvermoser@medigene.com (M.S.); monika.braun79@gmx.de (M.B.); d.brechtefeld@medigene.com (D.B.); christian.krendl@gmx.net (C.K.); c.geiger@medigene.com (C.G.); k.mutze@medigene.com (K.M.); m.buerdek@medigene.com (M.B.); swilde0979@gmail.com (S.W.); daniel.sommermeyer@gmx.de (D.S.); 2 Immunoanalytics-Research Group Tissue Control of Immunocytes (TCI), Helmholtz Zentrum München, 81377 Munich, Germany; noessner@helmholtz-muenchen.de; 3 Medigene AG, 82152 Planegg, Germany

**Keywords:** immunotherapy, cancer, TME, PD-1, TCR-T cells, PRAME

## Abstract

**Simple Summary:**

The development of effective adoptive T-cell therapies (ATCs) to treat solid tumors has several challenges: the choice of a suitable target antigen, the generation of a specific T-cell receptor (TCR) directed against this target, and the hostile tumor microenvironment (TME). The cancer/testis antigen Preferentially Expressed Antigen in Melanoma (PRAME) is a promising target for ATCs since it is highly expressed in several solid tumor indications, while its expression in normal tissues is mainly restricted to the testis. Using our well-established high throughput TCR generation and characterization process, we identified a highly potent PRAME-specific TCR. To convert the inhibitory PD-1 signal in T-cells to an activating signal, we designed a chimeric receptor consisting of the extracellular domain of PD-1 and the signaling domain of 4-1BB. Combining this PD1-41BB receptor with our lead PRAME-TCR generated a very promising T-cell product with a favorable preclinical in vitro safety profile and enhanced in vitro and in vivo anti-tumor efficacy.

**Abstract:**

The hostile tumor microenvironment (TME) is a major challenge for the treatment of solid tumors with T-cell receptor (TCR)-modified T-cells (TCR-Ts), as it negatively influences T-cell efficacy, fitness, and persistence. These negative influences are caused, among others, by the inhibitory checkpoint PD-1/PD-L1 axis. The Preferentially Expressed Antigen in Melanoma (PRAME) is a highly relevant cancer/testis antigen for TCR-T immunotherapy due to broad expression in multiple solid cancer indications. A TCR with high specificity and sensitivity for PRAME was isolated from non-tolerized T-cell repertoires and introduced into T-cells alongside a chimeric PD1-41BB receptor, consisting of the natural extracellular domain of PD-1 and the intracellular signaling domain of 4-1BB, turning an inhibitory pathway into a T-cell co-stimulatory pathway. The addition of PD1-41BB to CD8+ T-cells expressing the transgenic PRAME-TCR enhanced IFN-γ secretion, improved cytotoxic capacity, and prevented exhaustion upon repetitive re-challenge with tumor cells in vitro without altering the in vitro safety profile. Furthermore, a single dose of TCR-Ts co-expressing PD1-41BB was sufficient to clear a hard-to-treat melanoma xenograft in a mouse model, whereas TCR-Ts without PD1-41BB could not eradicate the PD-L1-positive tumors. This cutting-edge strategy supports development efforts to provide more effective TCR-T immunotherapies for the treatment of solid tumors.

## 1. Introduction

Successful treatment of cancer patients with T-cell receptor (TCR)-modified T-cells (TCR-Ts) requires a suitable target antigen, a TCR with high antigen sensitivity and specificity, and recipient T-cells with a strong capacity to kill tumor cells. In addition, for optimal treatment benefit, TCR-Ts must retain effector potential in a hostile tumor microenvironment (TME), comprised of immunosuppressive networks of soluble factors and cellular players that reduce T-cell infiltration and negatively influence the fitness, efficacy, and persistence of T-cells. Chronic exposure to antigen-depleted metabolic factors such as glucose, the presence of inhibitory cytokines such as IL-10 [1], and PD-L1 expression by tumor and stroma cells can drive T-cells into exhaustion and dysfunction [2]. T-cells found within tumors often express high levels of the PD-1 receptor [3] and are inhibited in multiple effector functions upon encounter with PD-L1-expressing tumor cells. Successful immunotherapies need to employ strategies to overcome inhibitory TMEs to prevent exhaustion and retain the function of transferred TCR-Ts. 

One strategy to enhance T-cell activities in the TME relies on the inhibition of negative PD-1/PD-L1 interactions using blocking antibodies [3]. This approach showed highly beneficial clinical outcomes in patients with solid cancer [4,5] but was often associated with drug-related immune-mediated toxicities [6,7]. The positive effects of blocking antibodies were also limited by poor penetration and heterogeneous distribution in solid tumors [8]. Furthermore, anti-PD-1/PD-L1 therapies only work well in tumors that are infiltrated by immune cells, specifically antigen-specific T-cells. Therefore, an advantageous alternative would be to directly arm TCR-Ts with a mechanism to interfere in PD-1 signaling and avoid the side-effects that occur with the systemic use of blocking antibodies. Previous studies [9,10,11,12] have explored this approach by expressing chimeric receptors composed of the extracellular domain of PD-1 and the signaling domain of the co-stimulatory molecule CD28, which is one of the primary targets of PD-1 signaling [13]. As an alternative, we evaluated a chimeric receptor that combines the natural extracellular PD-1-binding domain with the intracellular domain of 4-1BB (CD137), a stimulatory signaling module that is incorporated in several chimeric antigen receptors (CARs) that have shown strong clinical efficacy in CAR-T immunotherapy [14]. Equipping T-cells with a chimeric PD1-41BB co-stimulatory receptor directly coupled with a highly specific TCR offers a way to switch inhibitory PD-1/PD-L1 interactions into positive signals that enhance TCR-T function upon antigen encounter.

The Preferentially Expressed Antigen in Melanoma (PRAME) is a well-suited cancer/testis target antigen, having only a minor and selective expression pattern in healthy tissues [15,16]. In contrast, high levels of PRAME mRNA have been found in a number of different cancer indications, including melanoma [17,18], non-small cell lung cancer [19], multiple sarcoma subtypes [20,21], and epithelial ovarian cancer [22]. 

In this study, we isolated TCRs specific for PRAME in the context of HLA-A*02:01 with high antigen specificity and sensitivity that could trigger strong T-cell-mediated cytotoxic activity by tapping the non-tolerized repertoires of healthy volunteers, as previously described [23,24]. The potent lead PRAME-TCR was evaluated in side-by-side comparisons of TCR-Ts, with or without the chimeric PD1-41BB co-stimulatory receptor, using a set of preclinical efficacy and safety assays. TCR-Ts co-expressing PD1-41BB showed more potent responses directed against PRAME-positive, PD-L1-expressing tumor cells compared to TCR-Ts lacking PD1-41BB, without altering a favorable in vitro safety profile. The positive effect of PD1-41BB co-stimulation was very pronounced when TCR-Ts were analyzed using in vitro models mimicking the TME through repeated exposure to PD-L1-positive 3-dimensional (3D) tumor spheroids. Strikingly, a single dose of TCR-Ts co-expressing PD1-41BB and the PRAME-TCR was sufficient to clear tumors in vivo employing a mouse model in which transgenic TCR expression alone did not allow control of aggressive tumor growth. Thus, we demonstrated that the inclusion of the PD1-41BB co-stimulatory receptor in TCR-Ts is a promising strategy to develop more effective immunotherapies for the treatment of solid cancers.

## 2. Materials and Methods

### 2.1. Isolation of PRAME-Reactive T-Cell Clones

PRAME-reactive T-cells were isolated as previously described [23,24,25]. In brief, blood was drawn from healthy donors after obtaining informed consent in accordance with company and governmental guidelines and approved by the Ethics Commission of the Bavarian State Chamber of Medicine. Monocyte-derived mature dendritic cells (mDCs) were generated from peripheral blood mononuclear cells (PBMCs), as previously described [26]. In brief, PBMCs were isolated using Ficoll gradient with subsequent plate adherence. On day 8, mDCs were harvested and transfected with 20 µg in-vitro-transcribed (ivt) RNA encoding PRAME and 20 µg ivtRNA encoding HLA-A*02:01. Production of ivtRNA was performed using the mMESSAGE mMACHINE T7 Kit (Thermo Fisher Scientific, Waltham, MA, USA) and the RNeasy Mini Kit (Qiagen, Hilden, Germany), following the manufacturers’ instructions [23]. CD8+ T-cells were enriched by negative selection according to the manufacturer’s instructions (CD8+ T-Cell Isolation Kit, Milteny Biotec, Bergisch Gladbach, Germany) and co-cultured with the transfected mDCs at a ratio of 10:1. The cell culture was supplemented with 5 ng/mL IL-7 (PeproTech, Hamburg, Germany) at day 0 and propagated by the addition of 50 U/mL IL-2 (Novartis, Basel, Switzerland) every 2–3 days starting at day 1.

After 8–12 days, CD8+ T-cells specific for PRAME-SLL_425–433_ (SLLQHLIGL) in the context of HLA-A2 were identified by staining with custom-synthesized allophycocyanin (APC)- and phycoerythrin (PE)-labeled pentamers (ProImmune, Oxford, UK) according to the manufacturer’s instructions by including V450-labeled anti-human CD8 antibody (clone RPA-T8, Becton Dickinson Biosciences (BD), Franklin Lakes, NJ, USA). Cells were pre-gated on CD8, and multimer double-positive cells were sorted as single cells using a FACSAria Fusion flow cytometer (BD) or an SH800S cell sorter (Sony, SONY Biotechnology, San Jose, CA, USA) in 96-wells containing 200 µL re-stimulation cocktail consisting of irradiated PBMCs, irradiated LAZ388 cells, as well as the anti-CD3 antibody OKT-3 [23]. 

Two weeks after single-cell sorting, a fraction of growing T-cell clones was used to verify reactivity towards PRAME by co-culture with PRAME-transduced or untransduced (UT) lymphoblastoid cell lines (LCLs) endogenously expressing HLA-A*02:01 that were generated in-house. The release of IFN-γ was assessed as described below and T-cell clones displaying a PRAME-specific reaction pattern were further expanded by the addition of a fresh re-stimulation cocktail and analyzed.

### 2.2. TCR Sequencing, Cloning, and Transduction

TCR-α and TCR-β chains of PRAME-reactive T-cell clones were identified by next-generation sequencing using an established standard protocol for analysis with the MiSeq system (Illumina, San Diego, CA, USA). For sample preparation, the manufacturer’s recommendations were followed using the Dynabeads mRNA DIRECT Kit (Thermo Fisher), SMART Scribe reverse transcriptase (Takara Bio, Montain View, CA, USA), and the MiSeq V3 Kit (Illumina). The constant regions of all TCR chains were minimally murinized to increase the stability of the TCRs [27]. The corresponding TCR chains were linked by a P2A peptide linker [28], codon-optimized (GeneArt, Thermo Fisher) [29], and cloned into the pES.12-6 self-inactivating gamma-retrovirus vector. For initial testing of the different TCRs, production of viral supernatant and transduction of CD8+ T-cells was performed as previously described [24]. FACS of TCR-transduced T-cells was done by sorting up to 2 × 10^5^ cells and subsequent expansion of the cells by a rapid expansion protocol by addition of a re-stimulation cocktail, as described above [30]. The chimeric co-stimulatory receptor PD1-41BB, consisting of the extracellular and transmembrane region of PD-1 (CD279, amino acids (aa) 21–191) and the intracellular domain of 4-1BB (CD137, aa 214–255), was added to the vector 5′ of the TCR and separated by a T2A element. The sequence was codon-optimized (GeneArt, Thermo Fisher) [29], ordered from GeneArt (Thermo Fisher), and cloned into the pES.12-6 self-inactivating gamma-retrovirus vector. 

### 2.3. Effector Cell Preparation

Frozen vials of previously isolated CD8+ T-cells derived from fresh apheresis material (obtained from commercial suppliers; cell collection was performed in accordance with applicable current local regulations and requirements) were thawed and activated using anti-CD3 and anti-CD28 antibodies (Miltenyi Biotec). Cell culture medium (VLE-RPMI 1640 medium; PAN Biotech) was supplemented with 5 ng/mL IL-7 and 5 ng/mL IL-15 (CellGenix, Freiburg, Germany). Retroviral vector plasmids containing only the TCR as a transgene or the TCR in combination with PD1-41BB were co-transfected in HEK293FT cells with helper plasmids encoding Moloney MLV gag/pol and the GALV env gene to produce amphotropic retroviruses, as described previously [28]. The transfection was performed in T175 flasks (Greiner Bio-One, Frickenhausen, Germany) in a total volume of 35 mL DMEM High Glucose medium supplemented with 10% FCS, 1% NEAA, 1% L-glutamine, and 1% sodium-pyruvate (all Thermo Fisher); 48 h post-transfection, retroviral supernatants were harvested and 2 mL of filtered viral supernatant was added per well to Retronectin (Takara Bio)-coated 24-well plates that were centrifuged at 1000× *g* at 32 °C for 90 min. For transduction, 0.25–0.5 × 10^6^ CD8+ T-cells were added per 24-well. To reach higher transduction rates, the same T-cells were transduced in the same manner a second time approx. 6 h after the first transduction; 18 to 24 h after transduction, T-cells underwent a 10-day-phase of expansion after transfer into G-Rex flasks (Wilson Wolf, Saint Paul, MN, USA), during which they were supplied with fresh cytokines IL-7 and IL-15 (CellGenix) at a final concentration of 5 ng/mL twice a week. The cells were harvested from the G-Rex flasks, and transduction rates were determined before the cells were frozen and stored at −80 °C.

### 2.4. Cell Culture

T2 (DSMZ, Braunschweig, Germany), T2_PD-L1, LCLs (International Histo-compatibility Working Group, Fred Hutchinson Cancer Research Center, or generated in house by transduction of donor B-cells with EBV strain B95.8), MelA375 (ATCC, Manassas, VA, USA), MelA375_PD-L1, SKMel23 (Lonza, Basel, Switzerland), SKMel23_PD-L1, Mel624.38 (kind gift of M. Panelli, National Institutes of Health, Bethesda, MD, USA), Colo678 (DSMZ), and OVCAR-3 (ATCC) were maintained in RPMI 1640 containing 10% FCS, 1% non-essential amino acids (NEAA), 1% L-glutamine, and 1% sodium-pyruvate. NCI-H1650 (ATCC), NCI-H1755 (ATCC), and NCI-H1703 (ATCC) were cultivated in RPMI 1640 medium containing 10% FCS; 647-V (DSMZ), and SNB-19 (DSMZ) were cultivated in DMEM supplemented with 10% FCS, 1% NEAA, 1% L-glutamine and 1% sodium-pyruvate. OV7 (ECACC, Merck, Darmstadt, Germany) were cultivated in Ham’s F12 + DMEM (1:1) with 5% FCS, 1% L-glutamine, 0.5 µg/mL hydrocortisone, 10 µg/mL insulin, and 1% pen/strep. MCF-7 (ATCC) and MCF-7_PD-L1 were maintained in EMEM containing 10% FCS and 0.01 mg/mL insulin. SAOS-2 (ATCC) were cultivated in McCoy’s 5a Medium Modified containing 15% FCS. All cell lines were tested for mycoplasma negativity by PCR (Venor^®^GeM Classic, Minerva Biolabs, Berlin, Germany).

Primary HLA-A*02:01-positive normal human bronchial epithelial cells (NHBE, Lonza), human renal cortical epithelial cells (HRCEpC, PromoCell, Heidelberg, Germany), human renal epithelial cells (HREpC, PromoCell), renal proximal tubule epithelial cells (RPTEC, Lonza), normal human lung fibroblasts (NHLF, Lonza), human osteoblasts (HOB, Lonza), and induced pluripotent stem cell-derived iCell Cardiomyocytes (iCardio, FujiFilm CDI, Madison, WI, USA) were cultured according to the manufacturers’ instructions. Monocytes (Mono) were isolated from healthy donor PBMC; immature and mature dendritic cells (iDCs and mDCs, respectively) were generated from healthy donor monocytes [31].

### 2.5. Cell Surface Staining for Flow Cytometry and FACS

Cell surface marker staining was done according to standard protocols established previously [24,25]. The following fluorochrome-labeled antibodies were used according to manufacturers’ instructions: anti-human CD8-Horizon™ V450 (clone RPA-T8, BD), TRBV09-PE (TCR Vβ1, clone BL37.2, Beckman Coulter, Krefeld, Germany), PD-1-Alexa Fluor^®^ 647 (clone EH12.1, BD), and PD-L1-BV421 (clone MIH1, BD). Flow cytometric analysis was performed on a MACSQuant X (Miltenyi Biotec) or a BD LSRFortessa Flow Cytometer (BD). For data analyses, FlowJo 10.2 software (FlowJo, Ashland, OR, USA) was used. CD8+ T-cells specific for PRAME-SLL_425–433_ (SLLQHLIGL) in the context of HLA-A2 were identified by multimer staining. Fluorochrome-labeled pentamers were custom-synthesized (ProImmune), and staining was done according to the manufacturer’s instructions, also including staining for CD8. Cell sorting was conducted using a FACSAria Fusion flow cytometer (BD) or an SH800S cell sorter (Sony).

### 2.6. Quantitative REAL-Time PCR (qPCR)

To determine mRNA expression levels of the tumor antigen PRAME in different tumor cell lines, qPCR was used. For this, cDNA was generated from the tumor cell samples using the Transcriptor First Strand cDNA kit (Roche, Basel, Switzerland) and subsequently analyzed with the Real-Time PCR System Light Cycler 480 (Roche). PRAME-encoding cDNA was amplified using the forward primer 5′-agagcagtatatcgcccagt-3′ and the reverse primer 5′-ctcgggacttacatcggtca-3′. The house-keeping gene GUSB served as control and was used for normalization. 

### 2.7. Cytokine Release Assay

IFN-γ release of CD8+ T-cells in co-culture with different target cells was assessed in supernatant after 24 h of co-culture. Co-culture experiments were performed with different tumor cell lines, PRAME-SLL_425–433_ (SLLQHLIGL) peptide or homologous peptide-loaded T2 and T2_PD-L1 cells, LCL, or a panel of normal cells. Different transduction efficiencies of TCR-T preparations were adjusted with UT T cells to generate equal transduction rates for all cell preparations and ensure comparable total numbers of T-cells per well. Cytokine concentrations were determined using ELISA kits (Thermo Fisher; BD). The OD measurement was performed using a Multiskan Microplate-Photometer (Thermo Fisher). Background-corrected OD values were used for extrapolation using a third-degree polynomial.

### 2.8. Cytotoxicity Assay

Tumor cell lysis was assessed using an IncuCyte S3^®^ or Zoom^®^ device (Sartorius, Goettingen, Germany), following the manufacturer’s recommendations for real-time quantitative live-cell imaging. For this, tumor cells were transduced with NucLightRed or NucLightGreen (Sartorius) and seeded into 96-well flat-bottom plates 24 h prior to the addition of TCR- or TCR_PD1-41BB-transduced CD8+ T-cells at indicated E:T ratios. Lysis of tumor cells was monitored by scanning the plates at regular intervals (every 2 or 4 h). The number of NucLightRed or NucLightGreen-labeled tumor cells over time was calculated using IncuCyte software (V.2018A, V.2019B; Sartorius). 

To determine the cytotoxicity of transduced CD8+ T-cells in a 3-dimensional (3D) setting, NucLightRed- or NucLightGreen-labeled tumor cells were seeded in 96-well ultra-low attachment plates (ULA; Corning, New York, NY, USA) to form 3D spheroid structures. Three to five days after seeding the tumor cells, 2 × 10^4^ TCR+ CD8+ T-cells were added to the ULA plates containing the tumor spheroids. Tumor cell lysis was monitored by imaging the plates every 4 h using the IncuCyte S3^®^ or Zoom^®^ device (Sartorius). For repeated challenge of the transduced CD8+ T-cells with tumor cells, pre-seeded NucLight-Red or NucLightGreen-labeled tumor cell spheroids were transferred from ULA plates to the co-culture plates at different time points. Lysis of tumor cell spheroids was determined using the spheroid setting in IncuCyte software (V.2018A, V.2019B; Sartorius).

### 2.9. Analysis of Homologous Peptides

Homologous peptides with up to four differences to the original SLL (SLLQHLIGL) epitope were identified and assessed for their potential to bind to HLA-A2 using the prediction tool Expitope 2.0^®^ [32]. Peptides were ordered at peptides&elephants GmbH and subsequently tested for recognition by transduced and untransduced (UT) CD8+ T-cells when loaded onto T2_PD-L1 cells. For this, T2_PD-L1 cells were incubated with 10^−6^ M of the partially homologous peptides for 1 h at 37 °C. Afterwards, T2_PD-L1 cells were washed to remove unbound peptides and then transduced, and UT CD8+ T-cells were added in an effector to target the cell (E:T) ratio of 1:1. After 20 h of co-culture, supernatants were harvested, and secreted IFN-γ was determined using an IFN-γ ELISA.

String fragments containing 186 base pairs (bp) of the original gene sequence 5′ and 3′ of the respective peptide-coding region (midigene) or 30 bp of original gene sequence 5′ and 3′ of the respective peptide-coding region (minigene) were custom synthesized as codon-optimized DNA (GeneArt, Thermo Fisher). Up to five different minigene constructs were combined in one construct, separated by 6-histidine linkers. String fragments were cloned into the pGEM vector; 3′ of the midi- or minigenes of the HLA-A2-restricted mutated ASTN1 P1268L epitope (KLY) was included as a positive control antigen. SNB-19 cells were electroporated with 20 µg of respective ivtRNA using an exponential protocol at 300 V and 300 µF (Gene Pulser XCell, BioRad, Hercules, CA, USA) and then co-cultured with transduced and UT CD8+ T-cells with an E:T ratio of 1:1. After 20 h of co-culture, supernatants were harvested, and secreted IFN-γ was determined using an IFN-γ ELISA.

### 2.10. Single-Cell Secretome Analysis 

TCR-Ts with and without PD1-41BB were rested overnight in 50 U/mL IL-2 prior to starting the co-culture at an E:T ratio of 1:2 with MelA375_PD-L1 tumor cells seeded into poly-L-lysine coated wells of a 24-well plate. After 20 h of co-culture at 37 °C, T cells were harvested from co-cultures and enriched by MACS using an anti-CD8 antibody MACS kit (Miltenyi Biotec) according to the manufacturer’s instructions; 3 × 10^4^ total CD8+ T-cells were analyzed for single-cell poly-cytokine release using proteomic barcoded IsoCode Chips and the 32 protein Single-Cell PF Strength Panel (Human) kit on the IsoLight^®^ device (IsoPlexis, Branford, CT, USA) according to manufacturer’s instructions. Data processing was performed using IsoSpeak software (IsoPlexis, version 2.8.0.0).

### 2.11. Xenograft Models

The experiments were carried out with female NOD/Shi-Scid/IL-2Rγnull (NXG) mice (Janvier Labs, Le Genest-Saint-Isle, France). The animals were hosted by groups of 2–6 individuals in a ventilated cage (type II (16 × 19 × 35 cm, floor area = 500 cm^2^)). Mice received subcutaneous (s.c.) injections of 5 × 10^6^ PRAME-positive HLA-A*02:01-positive Mel624.38 or MelA375_PD-L1 tumor cells to establish s.c. xenografts; 7 days post tumor implantation for MelA375_PD-L1 and 14 days for Mel624.38, respectively, mice were randomized into different groups and received a single intravenous injection of TCR-Ts or an equal amount of UT T-cells. T-cell numbers were calculated based on T-cells expressing the PRAME-specific TCR, and 1 × 10^7^ TRBV09-positive CD8+ T-cells were injected per mouse. Transduction rates of TCR-Ts with and without PD1-41BB were adjusted by dilution with UT T-cells so that all mice in one experiment received the same total number of T-cells. Tumor volume was measured via calipers ~2 times a week by Length × (Width)^2^ × 0.52. All procedures and housing were performed at TransCure bioServices (Archamps, France) and have been reviewed and approved by the local ethics committee (CELEAG).

## 3. Results

### 3.1. Isolation of a High-Affinity PRAME-Specific TCR from a Non-Tolerized T-Cell Repertoire

**Figure 1:**
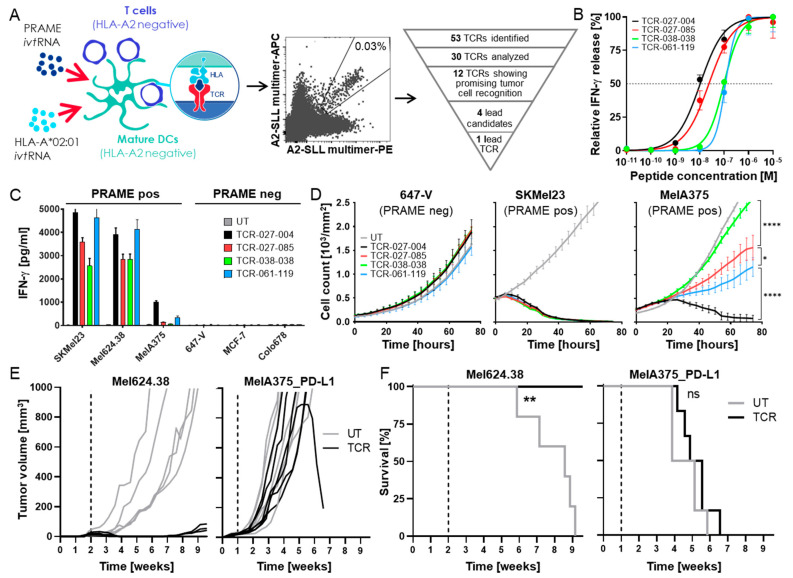
Isolation and selection of a lead PRAME-SLL-TCR from a non-tolerized T-cell repertoire. (**A**) CD8+ T-cells from HLA-A*02-negative healthy donors were stimulated using autologous monocyte-derived mature dendritic cells (mDCs) transfected with ivtRNA encoding PRAME and HLA-A*02:01. Dot blot shows staining with PE (*x*-axis) and APC (*y*-axis) labeled PRAME-multimers used for single-cell FACS. Percentage of double-positive cells is indicated. Promising TCRs were reconstructed and transferred into effector CD8+ recipient T-cells by retroviral transduction. Based on multi-parameter screening (triage process), four promising TCR candidates (TCR-027-004, TCR-027-085, TCR-038-038, and TCR-061-119) were selected from more than 30 analyzed specific TCRs. (**B**) Functional avidity of TCR-transgenic T-cells was analyzed in co-cultures with peptide-loaded T2 cells. Cells were cultured at an E:T ratio of 1:1. The graph shows nonlinear regression curves of relative IFN-γ values. Half maximal IFN-γ secretion is indicated by the dashed line at 50%. The data represent the means of duplicates measured at each peptide concentration. (**C**) IFN-γ concentrations released by the different TCR-Ts after co-culture with HLA-A*02-positive tumor cell lines that were either PRAME-positive (SKMel23, Mel624.38 and MelA375) or PRAME-negative (647-V, MCF-7 and Colo678). Untransduced (UT) T-cells served as negative control. IFN-γ values are shown as the mean of duplicates. Cells were cultured at an E:T ratio of 4:1 except for 647-V cells, which were cultured at an E:T ratio of 8:1. (**D**) Cytotoxicity of TCR-Ts expressing different PRAME-TCRs against fluorescently labeled HLA-A*02-positive tumor cell lines (PRAME-negative: 647-V; PRAME-positive: SKMel23, MelA375), assessed using a live-cell imaging system (IncuCyte^®^ ZOOM, Sartorius). Loss of red fluorescence visualized tumor cell apoptosis. UT effectors served as negative control. Cells were cultured at an E:T ratio of 4:1 except for 647-V cells, which were cultured at an E:T ratio of 8:1. Mean of cell counts using triplicates are shown over time. Statistical significance was calculated with one-way ANOVA with multiple comparisons (* *p* < 0.03, ** *p* < 0.002, **** *p* < 0.0001). Experiments in (**B**–**D**) were repeated three times with T-cells derived from different donors; data from one representative donor are shown. (**E**,**F**) 5 × 10^6^ Mel624.38 or MelA375_PD-L1 cells were injected s.c. into immunodeficient (NOD/Shi-scid/IL-2Rγnull) mice. Mice were treated on day 14 for Mel624.38 and day 7 for MelA375_PD-L1, respectively, with T-cells expressing TCR-027-004 or UT T-cells (5–6 mice per group) and (**E**) tumor growth and (**F**) survival of mice was followed until day 67 after tumor cell injection. Statistical significance between UT and TCR was calculated using the log-rank Mantel–Cox test (Mel624.38 *p* = 0.0018 and MelA375_PD-L1 *p* = 0.3597).

To obtain high-affinity HLA-A2-restricted TCRs specific for PRAME, we used our well-established high-throughput TCR discovery and selection procedures (Figure 1A). mDCs of healthy HLA-A*02-negative donors were transfected with ivtRNA encoding PRAME and HLA-A*02:01 and co-cultured with autologous CD8+ lymphocytes to induce HLA-A2-restricted, PRAME-specific T-cells from non-tolerized T-cell repertoires, as described previously [24,33]. After 8–12 days of co-culture, PRAME-specific CD8+ T-cells were sorted as single cells based on double-staining with two HLA-A2-PRAME-SLL_425–433_ multimers, each labeled with a different fluorochrome. Specific T-cell clones, identified in pre-screening for differential recognition of PRAME-positive vs. -negative cells, were analyzed using a broader tumor cell panel with multiple PRAME-negative or -positive tumor cell lines with varying levels of PRAME mRNA. Fifty-three unique TCR sequences that were identified among the T-cell clones showing specific recognition patterns underwent a triage process (Figure 1A). Thirty TCR sequences were cloned into a retroviral vector, transferred into recipient T-cells by retroviral transduction, and analyzed for expression of the TCRs on the cell surface, as well as for antigen-specific recognition of PRAME-positive, HLA-A2-positive tumor cell lines. Twelve TCRs showed good expression and recognized several tumor cell lines. These TCRs were tested for the recognition of a larger panel of tumor cell lines, their tumor cell killing capacity, as well as for potential cross-recognition of HLA-A2-presented peptides that are commonly found in healthy individuals. The four best TCRs in terms of specific recognition of PRAME-expressing HLA-A2-positive tumor cells and antigen specificity were tested in further activity and safety assessments. A hierarchy of functional avidity was identified among the four TCRs, with TCR-027-004 showing the greatest peptide sensitivity, followed by TCR-027-085, TCR-061-119, and TCR-038-038 (Figure 1B). T2 cells pulsed with irrelevant peptides were not recognized by the TCR-Ts. In addition, TCR-Ts released IFN-γ after stimulation with HLA-A*02:01/PRAME double-positive tumor cells but not after co-culture with PRAME-negative, HLA-A*02:01-positive tumor cells (Figure 1C). Differences among the four TCRs were most obvious after co-culture with the tumor cell line MelA375 that expressed only low levels of PRAME mRNA, whereby only TCR-Ts with TCR-027-004 released substantial amounts of IFN-γ. In addition, cytotoxic capacity analyzed via live-cell imaging showed that TCR-Ts with TCR-027-004 could eliminate MelA375 cells, whereas TCR-Ts expressing the other TCRs had only limited effects (Figure 1D). All TCR-Ts eliminated SKMel23 cells that expressed high PRAME levels, while PRAME-negative 647-V tumor cells were not recognized. TCR-027-004 was selected as the lead PRAME-TCR based on the highest functional avidity, best capacity to secrete IFN-γ after stimulation with tumor cells, and strongest triggering of cytotoxicity, particularly against tumor cells with low antigen levels.

The anti-tumor effect of TCR-Ts expressing TCR-027-004 was further analyzed in vivo in two models using immunodeficient NOD/Shi-scid/IL-2Rγnull mice bearing tumors derived either from the melanoma cell lines Mel624.38 or MelA375_PD-L1. Mel624.38-derived tumors grew relatively slowly and expressed high levels of PRAME but did not express PD-L1, whereas MelA375_PD-L1 cells grew far more aggressively, expressed substantially lower levels of PRAME, and had a high expression of PD-L1. A strong anti-tumor effect was observed in vivo against Mel624.38 cells; all mice treated with TCR-Ts on day 14 after tumor injection survived until the end of the experiment, and only small tumors were detectable in some of the mice at this timepoint (Figure 1E,F). In contrast, TCR-T treatment had no significant effect on the growth of MelA375_PD-L1 tumors compared to injection of untransduced (UT) Tßcells, and all mice had to be sacrificed between four and seven weeks (Figure 1E,F), indicating that a very potent PRAME-TCR was not sufficient to control growth in this very challenging PD-L1-positive tumor model.

### 3.2. Chimeric PD1-41BB Co-Stimulatory Receptor Improves Effector Functions of TCR-Ts

**Figure 2:**
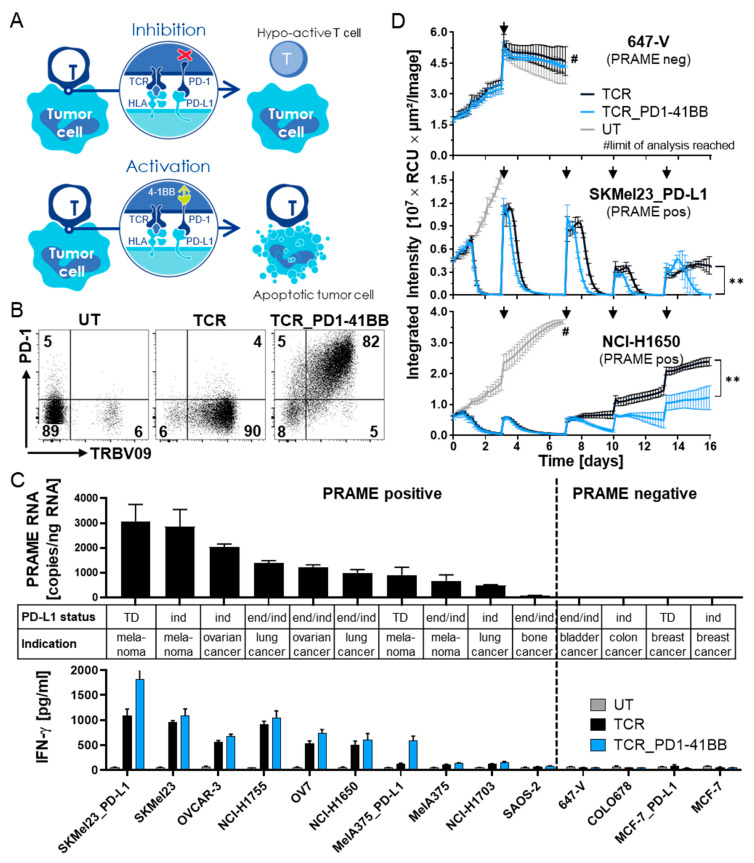
Co-expressing PD1-41BB on PRAME-TCR-Ts increases effector function in response to PRAME-positive tumor cell lines. (**A**) Interaction of wildtype PD1 expressed by T-cells with PD-L1 on tumor cells results in inhibition of T-cell activity and can lead to exhaustion and apoptosis. In contrast, the presence of the chimeric PD1-41BB co-stimulatory receptor on TCR-Ts can turn this inhibition into activation leading to improved effector functions, survival, and longevity. (**B**) Flow cytometric analysis of TCR-Ts with or without PD1-41BB for expression of the PRAME-TCR (TRBV09) (PE) and PD1-41BB (PD1) (Alexa Fluor-647). The analysis was repeated for transduced T-cells derived from 4 different donors. Data from one representative donor are shown. (**C**) Upper part: PRAME-RNA expression levels of tumor cell lines derived from different indications were analyzed by qPCR. Tumor cell lines are ordered from high to low expression from left to the right. Bars represent means of triplicates ± mean deviation. PD-L1 expression levels with and without IFN-γ treatment were analyzed by flow cytometry. PD-L1 status is indicated below the graph: (TD) transduced, (ind) IFN-γ inducible, (end) endogenously expressed. Lower part: TCR-Ts with or without PD1-41BB were co-cultured with the corresponding tumor cell lines at an E:T ratio of 1:1 and IFN-γ levels were determined 24 h after co-culture by ELISA. IFN-γ values are shown as the mean of triplicates ± mean deviation. (**D**) Cytotoxicity of TCR-Ts with or without PD1-41BB against red fluorescently labeled 3D tumor cell spheroids was monitored over 16 days using the IncuCyte S3^®^ device. T-cells were re-challenged with fresh spheroids on days 3, 7, 10 and 13, indicated by black arrows. PD-L1-transduced SKMel23 (PRAME-positive), endogenously PD-L1-expressing NCI-H1650 (PRAME-positive), and endogenously PD-L1-expressing 647-V (PRAME-negative) were used as target cells. UT T-cells served as control. Limit of analysis for UT T-cells was reached when red fluorescence could no longer be reliably calculated due to an excess of tumor cells in the well (#). Statistical significance was calculated from end-point values with unpaired t-test (TCR vs. TCR-PD1-41BB; *p* = 0.0076 (**) for SKMel23_PD-L1; *p* = 0.0081 (**) for NCH-H1650). Experiments in (**B**–**D**) were repeated four times with T-cells derived from different donors; data with cells from one representative donor are shown.

To overcome the inhibitory effect of the PD-1/PD-L1 axis, the PRAME-specific TCR-027-004 was co-expressed with a chimeric PD1-41BB receptor in TCR-Ts, whereby PD-L1 interaction with the chimeric receptor should turn the inhibitory signal into additional T-cell co-stimulation (Figure 2A). CD8+ TCR-Ts with TCR-027-004 alone (TCR) or combined with PD1-41BB (TCR_PD1-41BB) were generated, and surface expression of one or both receptors was confirmed by flow cytometry (Figure 2B). 

HLA-A*02:01-positive tumor cell lines with varying levels of PRAME mRNA and derived from different indications were used as target cells to analyze the effector functions of the two TCR-T populations in vitro (Figure 2C). PRAME protein expression in target cells was also confirmed by Western blot. Cell surface expression of PD-L1 on tumor cell lines was determined via flow cytometry. While some tumor cell lines showed endogenous PD-L1 expression (end), expression of PD-L1 could be induced in other cell lines by IFN-γ (ind). Some tumor cell lines were transduced with PD-L1 (TD) to ensure stable cell surface expression. Co-expression of PD1-41BB with the PRAME-specific TCR on CD8+ TCR-Ts enhanced the release of IFN-γ in response to PD-L1-positive tumor cells (Figure 2C). UT CD8+ T-cells serving as negative control did not release any IFN-γ upon tumor cell stimulation. Expression of PD-L1 on the target cells did not influence TCR specificity since PRAME-negative tumor cells with either endogenous or transgenic PD-L1 expression were not recognized by either of the TCR-T populations. 

To mimic several parameters of TMEs, 3D tumor cell spheroids were created from representative tumor cell lines with different levels of PRAME and PD-L1 expression. The spheroids were added to cultures of the two TCR-T populations at five consecutive timepoints. Tumor cell growth was monitored over 16 days using live-cell imaging. In this challenging tumor recognition model in vitro, TCR-Ts co-expressing PD1-41BB with the PRAME-TCR were more effective in controlling tumor growth compared to TCR-Ts without PD1-41BB, especially after several re-exposures to tumor spheroids (Figure 2D and Figure S1). PRAME-negative, PD-L1-positive 647-V cells were not targeted by the TCR-Ts. These in vitro activity experiments revealed that TCR-Ts co-expressing PD1-41BB exhibited stronger effector functions and retained better cytotoxic function upon repeated antigen exposure, even after prolonged contact with PD-L1-positive tumor spheroids.

### 3.3. TCR-T In Vitro Safety Profile Is Not Changed by Co-Expression of PD1-41BB

**Figure 3:**
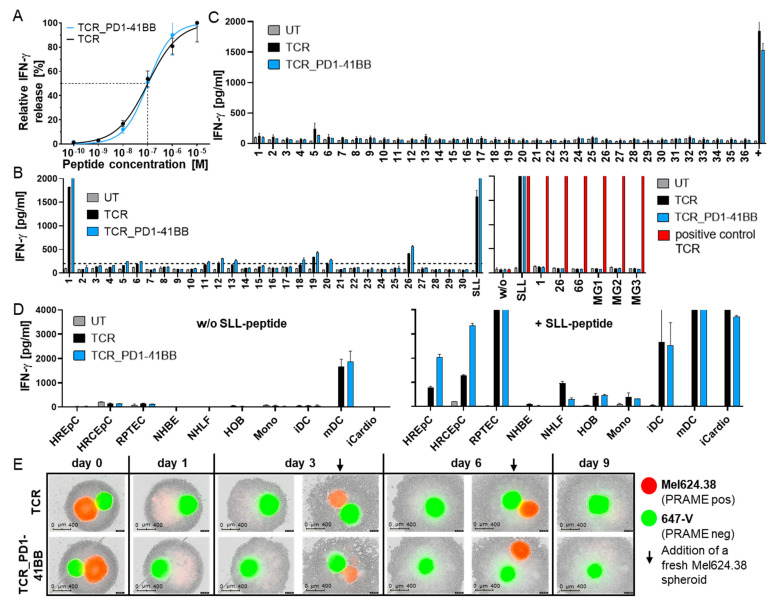
Co-expression of PD1-41BB does not change the in vitro safety profile of the lead PRAME-SLL-TCR. (**A**) Functional avidity of CD8+ TCR-Ts, with or without PD1-41BB, following stimulation with PD-L1-transduced T2 cells loaded with graded amounts of PRAME peptide (10^−10^ M–10^−5^ M). Cells were cultured at an E:T ratio of 1:1. The graph shows nonlinear regression curves of relative IFN-γ release. Peptide concentration needed for half maximal IFN-γ secretion is indicated by the dashed line. The data represent means of duplicates measured at each peptide concentration. (**B**) Left part: The recognition of 191 peptides with high sequence homology with the specific PRAME-SLL peptide by CD8+ TCR-Ts with or without PD1-41BB was analyzed. Peptides were loaded onto T2_PD-L1 cells at a concentration of 10^−6^ M. IFN-γ release data for 30 peptides are shown as an example; data for all peptides are shown in Figure S2. Right part: PRAME-negative, PD-L1-expressing SNB-19 cells were transfected with RNA constructs encoding either a midigene (~400 bp) coding for single peptides (SLL, 1, 26, 66) or minigene (~90 bp per peptide) constructs (MG) coding for up to five variant peptides and co-cultured with effector cells. All constructs included an epitope recognized by a positive control TCR. UT T-cells served as negative control. For read-out, supernatants were harvested after 20 h and analyzed by IFN-γ ELISA. The data represent means of duplicates. (**C**) IFN-γ released by CD8+ TCR-Ts with or without PD1-41BB co-cultured with 36 LCLs covering frequent HLA-A, -B, and -C alleles. UT CD8+ T-cells served as negative control, and PRAME-SLL-peptide-loaded HLA-A*02:01 positive LCLs were used as positive control. (**D**) IFN-γ released by CD8+ TCR-Ts with or without PD1-41BB co-cultured with normal cells derived from critical healthy tissues. As positive controls, cells were loaded with PRAME SLL-peptide. HREpC: human renal epithelial cells, HRCEpC: human renal cortical epithelial cells, RPTEC: renal proximal tubule epithelial cells, NHBE: normal human bronchial epithelial cells, NHLF: normal human lung fibroblasts, HOB: human osteoblasts, Mono: monocytes, iDC: immature dendritic cells, mDC: mature dendritic cells, iCardio: iCell Cardiomyocytes. The data represent means of duplicates. (**E**) Cytotoxicity of CD8+ TCR-Ts with or without PD1-41BB against PRAME-positive PD-L1-negative (red) and PRAME-negative PD-L1-positive (green) 3D tumor cell spheroids. Fresh PRAME-positive Mel624.38 spheroids (red) were added to the co-cultures on days 3 and 6, indicated by black arrows. For days 3 and 6 pictures, before and after adding fresh tumor cell spheroids are shown. Experiments in A–E were repeated at least three times with T-cells derived from different donors; data with cells from one representative donor are shown.

Several assays were performed in vitro to confirm that increased reactivity of TCR-Ts co-expressing PD1-41BB had no impact on their safety profile. Functional avidity of TCR-Ts, with or without PD1-41BB, was analyzed in TCR-T co-cultures with peptide-loaded T2 cells over-expressing PD-L1. Co-expression of PD1-41BB did not alter functional avidity, measured as the peptide concentration needed for half-maximal relative IFN-γ release (Figure 3A), although the levels of IFN-γ released by TCR-Ts co-expressing PD1-41BB were higher.

Potential off-target toxicities were assessed by screening responses to 191 peptides with sequence homology to PRAME-SLL that had been selected using the Expitope 2.0^®^ tool [32]. Peptides were loaded at high peptide concentrations onto T2_PD-L1 cells and tested in co-cultures with PRAME-TCR-expressing TCR-Ts with or without PD1-41BB. Fourteen peptides stimulated IFN-γ release above background levels (200 pg/mL), irrespective of the presence of PD1-41BB, but only peptide #1 was recognized at a level comparable to the PRAME-SLL peptide (Figure 3B and Figure S2). To clarify whether variant peptides would be endogenously processed by target cells and presented at levels adequate to stimulate TCR-T responses, the sequences of stimulatory peptides were cloned either as midigenes (~400 bp per construct, each encoding a single peptide: PRAME-SLL, #1, #26, #66) or as a construct of minigenes (MG1-3: ~90 bp per peptide, encoding up to five peptides per construct). PD-L1-positive, PRAME-negative SNB-19 cells were transfected individually with the different constructs. Successful transfection, antigen processing, and surface HLA-A2-presentation from the constructs were confirmed by showing that SNB-19 cells, transfected with each mini- or midigene construct, were recognized by an unrelated control TCR whose specific epitope was included in every construct. PRAME-transfected SNB-19 cells triggered TCR-T responses, but no variant peptide encoded by any of the constructs was recognized by the TCR-Ts, irrespective of PD1-41BB co-expression (Figure 3B). This demonstrated that the high specificity of the PRAME-TCR was not altered by PD1-41BB co-expression in TCR-Ts.

Cross-recognition of naturally occurring endogenous peptides presented by a variety of HLA molecules was tested using a library of LCLs carrying frequent HLA-A, -B, and -C alleles. No off-target toxicity due to HLA allo-cross-recognition was seen (Figure 3C). PRAME-peptide loaded HLA-A*02:01-positive LCLs serving as a positive control were recognized by both TCR-Ts. Additionally, one unloaded HLA-A*02:01-positive LCL (#5) that led to moderate IFN-γ release after co-culture with both TCR-Ts was found to naturally express low levels of PRAME mRNA, as determined by qPCR (data not shown). This showed the high sensitivity of this assay.

The impact of PD1-41BB co-expression on TCR-T recognition of critical healthy tissues was assessed by comparing both TCR-Ts in co-cultures using a panel of normal cells. Among the numerous cell types studied, only PRAME-positive mDCs were recognized by TCR-Ts, as previously observed for other PRAME-specific T-cells [34], irrespective of PD1-41BB expression. In contrast, PRAME-negative mDC progenitors (monocytes and immature DCs) did not trigger IFN-γ release by either TCR-T population (Figure 3D). Other normal cells only induced IFN-γ release by TCR-Ts after exogenous loading with PRAME-SLL peptide. Therefore, off-target recognition of critical healthy tissues was not observed with PRAME-specific TCR-Ts irrespective of PD1-41BB co-expression.

To analyze whether off-target toxicity would occur against PRAME-negative, PD-L1-positive cells by antigen-activated TCR-Ts, simultaneous cytotoxicity directed against mixtures of PRAME-positive and PRAME-negative target cells was studied; 3D spheroids of PRAME-negative, PD-L1-positive 647-V cells and PRAME-positive, PD-L1-negative Mel624.38 cells were co-cultured with both TCR-T populations. Even after the sequential addition of fresh PRAME-positive spheroids, TCR-Ts remained strictly antigen-dependent and showed no cytotoxic effects against PRAME-negative spheroids that expressed PD-L1 (Figure 3E). This demonstrated that innocent bystander killing of PRAME-negative cells was not triggered in the TCR-Ts through PD1-41BB alone, and co-stimulation was coupled to antigen-specific TCR recognition. Importantly, in most co-culture experiments, the number of TCR-T cells slightly decreased during several days of co-culture with PRAME-expressing tumor cells independently of whether the TCR-Ts expressed or did not express PD1-41BB. There was no sign of uncontrolled growth of TCR-Ts expressing PD1-41BB. However, when TCR-Ts co-expressed PD1-41BB, increased survival of the T-cells was observed in co-cultures with PRAME-positive tumor cells expressing PD-L1 (data not shown). This combination of in vitro assays confirmed the excellent safety profile of the PRAME-TCR and did not reveal any alterations in the safety characteristics of TCR-Ts co-expressing PD1-41BB.

### 3.4. TCR-Ts Co-Expressing the Chimeric PD1-41BB Co-Stimulatory Receptor Reject Tumors In Vivo and Have a Poly-Cytokine Profile

**Figure 4:**
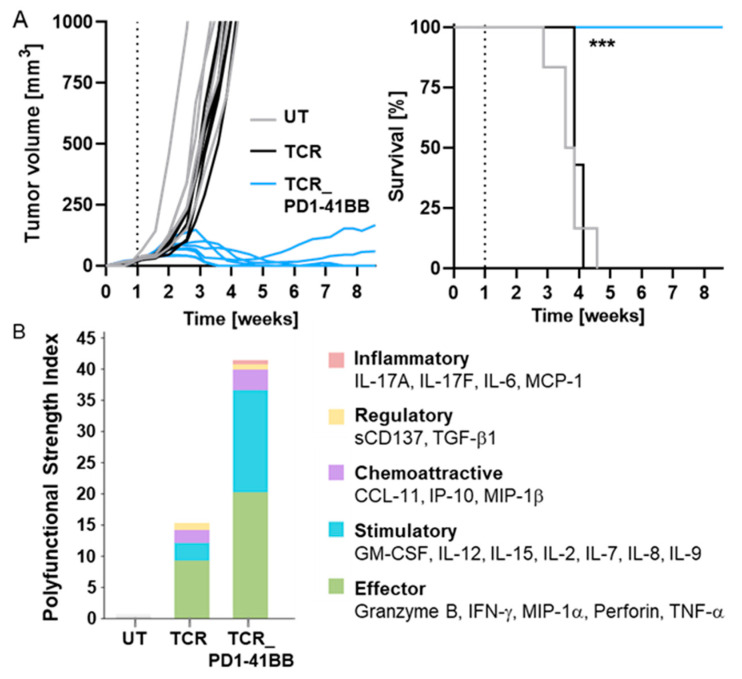
TCR-Ts co-expressing PD1-41BB reject tumor cells in vivo and show a superior poly-cytokine profile. (**A**) 5 × 10^6^ MelA375_PD-L1 cells were injected s.c. into immunodeficient (NOD/Shi-scid/IL-2Rγnull) mice. Mice were treated on day seven either with 10 × 10^6^ PRAME-TCR-positive CD8+ TCR-Ts with (7 mice) or without (7 mice) PD1-41BB (14 × 10^6^ cells in total per mouse) or 14 × 10^6^ UT T-cells (6 mice), and tumor growth (left panel) and survival (right panel) was followed for 60 days. Statistical significance between TCR and TCR_PD1-41BB was calculated using the log-rank Mantel–Cox test (*p* = 0.0003, ***). (**B**) Co-cultures of MelA375_PD-L1 cells and TCR-Ts, with and without PD1-41BB, were performed in poly-L-lysine pre-coated 24 well plates. After 20 h, T-cells were harvested, enriched for CD8 expression by MACS, and applied onto IsoCode Chips. Polyfunctional strength index (PSI), which combines the analysis of identity and quantity of secreted cytokines/proteins, is depicted. Analysis is based on all cytokines/proteins which were detected by the Single-Cell PF Strength Panel kit designed for 32 different proteins. Experiment was repeated two times with T-cells derived from different donors. Data from one representative donor are shown.

Whether PD1-41BB co-stimulation of TCR-Ts would drive enhanced efficacy in vivo was studied in a difficult-to-treat melanoma xenograft mouse model (Figure 1E). Human TCR-Ts were transferred into immunodeficient mice bearing s.c. MelA375_PD-L1 tumors that expressed low levels of PRAME and high levels of PD-L1. On day 7 after tumor cell injection, a single dose of TCR-Ts, with or without PD1-41BB, was applied intravenously; UT T-cells derived from the same donor served as controls. Treatment with TCR-Ts without PD1-41BB had no significant effect on the survival of mice compared to treatment with UT T-cells (Figure 4A), confirming the previous observations (Figure 1E,F). Remarkably, TCR-Ts co-expressing PD1-41BB were able to mediate complete tumor rejection. Five of seven mice had no measurable tumor at the end of the experiment, and all mice treated with TCR-Ts expressing PD1-41BB survived until the end of the experiment, whereas all mice in the other two groups had to be sacrificed between three and five weeks due to large tumor burdens. These data showed that PD1-41BB co-stimulation resulted in highly improved efficacy that enabled TCR-Ts to effectively eliminate aggressively growing tumors in vivo that expressed high levels of PD-L1 despite only expressing low levels of PRAME.

Samples of the TCR-Ts that were applied in vivo were analyzed for polyfunctionality on a single cell level after in vitro stimulation with MelA375_PD-L1 tumor cells using the IsoPlexis technology platform. TCR-Ts co-expressing PD1-41BB displayed a significantly higher polyfunctional strength index (PSI) compared to TCR-Ts without PD1-41BB (Figure 4B). The increased secretion of effector and stimulatory proteins following PD1-41BB co-stimulation likely contributed to the superior anti-tumor efficacy that was seen in vivo. Notably, regulatory and inflammatory cytokines were released at minor levels only, which reduces the likelihood of autoinflammation due to PRAME TCR-T cells expressing PD1-41BB.

## 4. Discussion

Adoptive T-cell immunotherapy is a promising treatment approach for a variety of tumor indications [35]. Tumor-antigen-specific T-cells have the potential to provide long-term protection against tumor relapse, but response rates of adoptive T-cell immunotherapies for solid tumors remain low [36,37]. When targeting solid tumors, T-cells are faced with hostile TMEs that result in T-cell dysfunction. Besides inhibitory cytokines and depleted metabolic factors, the inhibitory checkpoint PD-1/PD-L1 axis reduces T-cell infiltration and negatively influences efficacy, fitness, and persistence of TCR-Ts in many types of cancer. Thus, new strategies are needed to overcome inhibitory TMEs to prevent the exhaustion of transferred TCR-Ts.

Immune checkpoints such as PD-1 play a complex role in the immune system [7]. Activated T-cells express PD-1, which can bind to PD-L1 that is upregulated by IFN-γ on normal tissues during inflammatory responses to pathogen infections [38]. This mechanism protects normal tissues and prevents autoimmunity. However, PD-L1 is also expressed by many tumors, leading to an inhibitory TME for tumor-infiltrating T-cells [39,40,41,42,43].

This inhibitory axis in the TME can be overcome by the application of PD-1 or PD-L1 blocking antibodies [44]. Nevertheless, reaching sufficient levels of blocking antibodies for improved anti-tumor responses can be challenging, and the systemic antibody administration can lead to adverse events due to the pivotal role that the PD-1/PD-L1 pathway simultaneously plays in preventing autoimmunity [4,45]. 

Many cancer patients do not benefit from anti-PD-1 monotherapy [46], in some cases explained by the paucity of tumor-specific T-cells in the tumors. Combining immunomodulatory antibodies with adoptive cell therapies can remedy the paucity of T-cells and has shown promising preliminary clinical results [36]. To avoid systemic administration of PD-1 antibodies, PD-1 depletion/knock-out using gene editing in transferred anti-tumor T-cells or TCR-equipped T-cells is considered a strategy to locally block T-cell inhibition [40,47]. For TCR-Ts, an additional knock-out of the endogenous TCR might be needed since control by PD-1/PD-L1 in peripheral healthy tissues is no longer possible in the absence of PD-1 expression by the T cells [48]. 

Chimeric receptors that couple PD-1 to a cytoplasmic activating signaling domain not only prevent inhibition during PD-L1 interaction but additionally deflect the signaling into co-stimulation. Co-expression of a chimeric PD1-CD28 receptor on CAR-Ts yielded more potent effects in solid tumors than seen with antibody-based PD-1 blockade [12]. Similarly, the incorporation of a chimeric PD-1 receptor in TCR-Ts could upgrade the functional activity of low-avidity TCR-Ts [13]. Most recently, it was shown that the inclusion of the 4-1BB co-stimulatory domain in the signaling tails of CARs increased CAR-T persistence and resulted in better expansion compared to CAR-Ts with the CD28-domain [49]. Taking these observations into account, we explored the use of a chimeric PD1-41BB receptor, consisting of the extracellular and transmembrane domains of PD-1 and the intracellular co-stimulatory domain of 4-1BB, with the aim of turning inhibitory PD-1/PD-L1 interactions into enhanced T-cell co-stimulation.

Our very potent high-affinity lead PRAME-specific TCR could broadly recognize tumor cell lines derived from different cancer indications with high specificity. Inclusion of PD1-41BB in these TCR-Ts enhanced IFN-γ responses to PD-L1-positive tumor cells but did not change functional avidity and the favorable in vitro safety profile of the TCR. Among cells derived from healthy tissue, only mDCs triggered secretion of IFN-γ in TCR-Ts due to endogenous PRAME expression [34], which was not enhanced by co-expression of the PD1-41BB receptor. Since progenitor monocytes and immature DCs were not recognized by the TCR-Ts, monocyte-derived mDCs can be replenished, mitigating any safety concerns.

PD1-41BB co-expressing TCR-Ts showed superior tumor cell killing compared to TCR-Ts without PD1-41BB in a tumor-mimicking in vitro situation of repeated T-cell challenge with 3D tumor cell spheroids expressing PRAME and PD-L1. Moreover, a single dose of PD1-41BB-expressing PRAME T-cells achieved in vivo tumor control of an aggressively growing xenograft with low antigen density and high expression of PD-L1 that was resistant to eradication by T-cells only expressing the highly potent PRAME TCR. The activity of TCR-Ts in these mice was fully dependent upon their own inherent properties since human cytokines were not supplied, and murine cytokines cannot serve as a substitute. The high number of T-cells with polyfunctionality might have contributed to the remarkable capacity in vivo in which mice received no supplementary human cytokines. 

While strongly enhancing T-cell function, the activity of the PD1-41BB co-stimulatory receptor was strictly coupled to the TCR signal and PD-L1 interaction, as PD-L1 binding to PD1-41BB did not activate cytotoxicity in the absence of a TCR signal. Thereby, PD1-41BB co-stimulation is provided in the context of tumor recognition in the TME, when and where it is required to support T-cell function. By converting the inhibitory signal of PD-L1 expression by tumor cells into co-stimulation, PD-L1 no longer supports immune evasion but instead drives enhancement of TCR-T efficacy and contributes to tumor cell elimination. 

## 5. Conclusions

The HLA-A2-restricted, PRAME-specific TCR was shown to recognize a broad panel of tumor cell lines representing different cancer indications. It had a very favorable preclinical safety profile in vitro, as determined through the assessment of critical healthy tissues. Although TCR-Ts expressing this potent TCR could effectively control PD-L1-negative tumor cells with adequate levels of PRAME in an in vivo model, they failed when confronted with tumors characterized by low PRAME and high PD-L1 expression. This deficiency was overcome by the provision of the chimeric PD1-41BB co-stimulatory receptor alongside the potent TCR, augmenting and prolonging T-cell activity and fitness upon multiple exposures to tumor spheroids in vitro and enabling the control of aggressive tumors in vivo. PD1-41BB is a promising tool to be integrated into the engineering process of TCR-Ts to overcome the prevalent PD-1/PD-L1 inhibition in the TME of many solid cancers while bypassing the negative side-effects associated with the systemic application of blocking antibodies. 

## 6. Patents

N.S., I.F., M.S., C.K., S.W., and D.S. are designated as inventors on two patent applications (PRAME-SLL: WO2021/099360, PRAME-SLL/PD1-41BB EP21172722) related to this work that have been filed by Medigene Immunotherapies GmbH. 

E.N. is inventor and patent holder on PD1-41BB (WO2017/162797) filed by Helmholtz Zentrum München.

## Data Availability

Data are available upon reasonable request. Data is available from D.J.S.

## Supplementary Materials

The following supporting information can be downloaded at: https://www.mdpi.com/article/10.3390/cancers14081998/s1, Figure S1. Cytotoxicity of TCR-Ts, with or without PD1-41BB, against red fluorescently labelled 3D tumor cell spheroids was monitored over 16 days using the IncuCyte S3® device. T cells were re-challenged with fresh spheroids on day 3, 7, 10 and 13 indicated by black arrows. PD-L1-transduced SKMel23 (PRAME-positive), endogenously PD-L1-expressing NCI-H1650 (PRAME-positive) were used as target cells. For day 3, 7, 10 and 13 pictures before and after adding fresh tumor cell spheroids are shown., Figure S2. Variant peptides were loaded onto T2_PD-L1 cells at a peptide concentration of 10-6 and recognition by TCR-Ts without or with PD1-41BB was tested. UT T cells served as negative control. The specific PRAME-SLL peptide and an irrelevant peptide were used as controls. For read-out, supernatants were harvested after 20 h and analyzed by IFN-γ ELISA. The data represent means of duplicates. Peptides that were recognized above the background (200 pg/mL) are marked. Experiments were performed with T cells derived from four different donors; data with cells from one representative donor are shown.
