# Supplementary material for: T-Cells Expressing a Highly Potent PRAME-Specific T-Cell Receptor in Combination with a Chimeric PD1-41BB Co-Stimulatory Receptor Show a Favorable Preclinical Safety Profile and Strong Anti-Tumor Reactivity
Source: Cancers (Basel). 2022 Apr 14;14(8):1998. doi: 10.3390/cancers14081998 (PMC9030144; doi:10.3390/cancers14081998)
Supplement: Supplementary file 1 [file cancers-14-01998-s001.zip › cancers-1603146-supplementary.pdf]

Supplementary Figures

T-Cells Expressing a Highly Potent PRAME-Specific T-Cell Receptor in Combination with a Chimeric PD1-41BB Co-Stimulatory Receptor Show a Favorable Preclinical Safety Profile and Strong Anti-Tumor Reactivity

Nadja Sailer, Ina Fetzer, Melanie Salvermoser, Monika Braun, Doris Brechtefeld, Christian Krendl, Christiane Geiger, Kathrin Mutze, Elfriede Noessner, Dolores J. Schendel, Maja Bürdek, Susanne Wilde and Daniel Sommermeyer

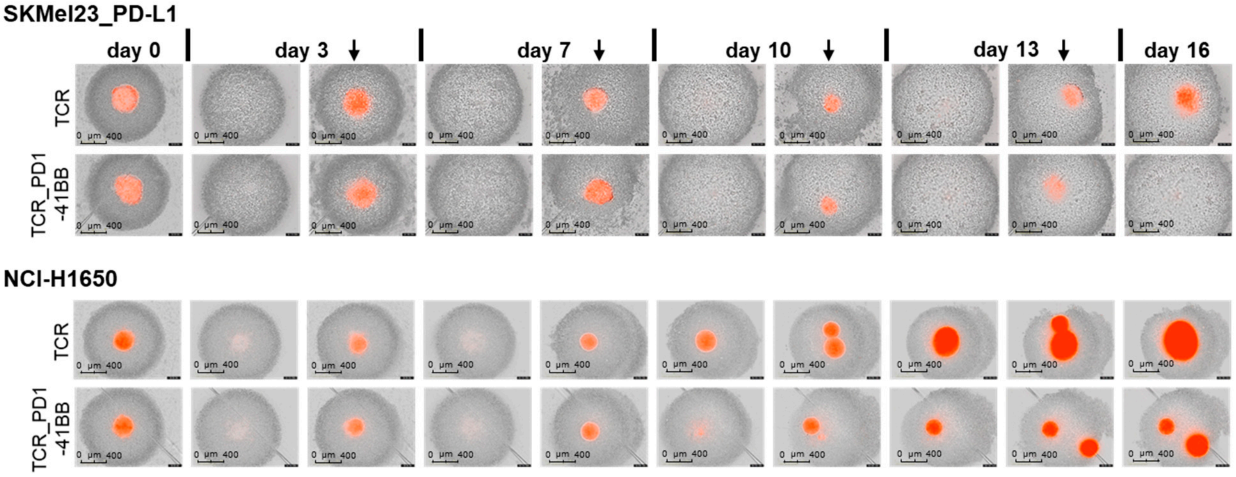

Figure S1. Cytotoxicity of TCR-Ts, with or without PD1-41BB, against red fluorescently labelled 3D tumor cell spheroids was monitored over 16 days using the IncuCyte S3® device. T cells were re-challenged with fresh spheroids on day 3, 7, 10 and 13 indicated by black arrows. PD-L1-transduced SKMel23 (PRAME-positive), endogenously PD-L1-expressing NCI-H1650 (PRAME-positive) were used as target cells. For day 3, 7, 10 and 13 pictures before and after adding fresh tumor cell spheroids are shown.

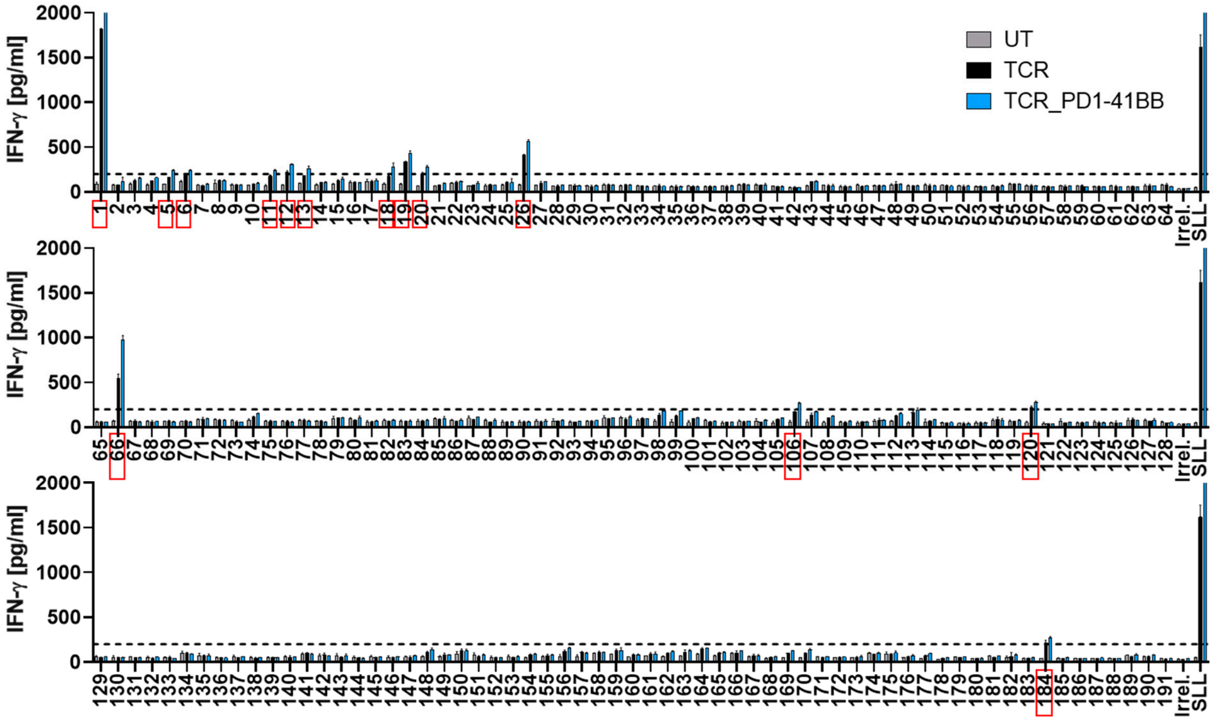

Figure S2. Variant peptides were loaded onto T2_PD-L1 cells at a peptide concentration of 10^{-6} and recognition by TCR-Ts without or with PD1-41BB was tested. UT T cells served as negative control. The specific PRAME-SLL peptide and an irrelevant peptide were used as controls. For read-out, supernatants were harvested after 20 h and analyzed by IFN-γ ELISA. The data represent means of duplicates. Peptides that were recognized above the background (200 pg/ml) are marked. Experiments were performed with T cells derived from four different donors; data with cells from one representative donor are shown.
